# The Preventive Effect of Low-Molecular Weight Oyster Peptides on Lipopolysaccharide-Induced Acute Colitis in Mice by Modulating Intestinal Microbiota Communities

**DOI:** 10.3390/foods13152391

**Published:** 2024-07-29

**Authors:** Qihang Wu, Haisheng Lin, Weiqiang Shen, Wenhong Cao, Xiaoming Qin, Jialong Gao, Zhongqin Chen, Huina Zheng, Saiyi Zhong, Haoyang Huang

**Affiliations:** 1 College of Food Science and Technology, Guangdong Ocean University, Zhanjiang 524088, China; 2112203066@stu.gdou.edu.cn (Q.W.); shenweiqiang1@stu.gdou.edu.cn (W.S.); cwenhong@gdou.edu.cn (W.C.); xiaoming0502@21cn.com (X.Q.); gaojl@gdou.edu.cn (J.G.); chenzhongqin@gdou.edu.cn (Z.C.); zhenghn@gdou.edu.cn (H.Z.); zsylxc@126.com (S.Z.); huanghao7@stu.gdou.edu.cn (H.H.); 2 National Research and Development Branch Center for Shellfish Processing (Zhanjiang), Guangdong Ocean University, Zhanjiang 524088, China; 3 Guangdong Provincial Key Laboratory of Aquatic Product Processing and Safety, Guangdong Ocean University, Zhanjiang 524088, China; 4 Guangdong Province Engineering Laboratory for Marine Biological Products, Guangdong Ocean University, Zhanjiang 524088, China

**Keywords:** colitis, anti-inflammatory, intestinal microbiota, oyster peptides

## Abstract

Colitis causes inflammation, diarrhoea, fever, and other serious illnesses, posing a serious threat to human health and safety. Current medications for the treatment of colitis have serious side effects. Therefore, the new strategy of creating a defence barrier for immune function by adding anti-inflammatory foods to the daily diet is worth advocating for. Low-molecular weight oyster peptides (LOPs) are a natural food with anti-inflammatory activity extracted from oysters, so intervention with LOPs is likely to be an effective preventive solution. The aim of this study was to investigate the preventive effect of LOPs on lipopolysaccharide (LPS)-induced acute colitis inflammation in mice and its underlying mechanism. The results showed that LOPs not only inhibited the colonic histopathy in mice induced by LPS-induced inflammation but also reduced the inflammatory response in the blood. In addition, LOPs significantly increased the number of beneficial bacteria (*Alistipes*, *Mucispirillum*, and *Oscillospira*), decreased the number of harmful bacteria (*Coprobacillus*, *Acinetobater*) in the intestinal microbiota, and further affected the absorption and utilisation of short-chain fatty acids (SCFAs) in the intestinal tract. In conclusion, dietary supplementation with LOPs is a promising health-promoting dietary supplement and nutraceutical for the prevention of acute colitis by reducing the inflammatory response and modulating the intestinal microbial communities.

## 1. Introduction

Colitis is the most common digestive system disease, and it is an inflammation of the intestinal mucosa caused by various factors. It is more prevalent in underdeveloped regions and areas with poor sanitation [1]. Clinical symptoms include nausea, vomiting, abdominal pain, diarrhoea, and fever [2]. In severe cases, it can lead to dehydration or shock. Prolonged localised inflammation can also result in systemic inflammation, which can affect organs that were previously unaffected [3]. Furthermore, patients diagnosed with colitis are at an elevated risk of developing rectal tumours and cancer [4]. The current treatment for colitis includes medications such as aminosalicylic acid preparations, glucocorticoids, and immunosuppressants. These drugs have been demonstrated to be effective in the treatment of colitis. However, they may also cause the risk of allergic reactions, bone marrow suppression, and osteoporosis in the body [3,5,6]. The development of food-derived natural products to prevent or reduce the risks associated with colitis has received increasing attention [7,8,9]. 

The ocean covers more than 70% of the Earth’s surface and is rich in functional active substances. Marine active peptides, in particular, have garnered widespread attention for their potential to promote health and reduce disease risk [10]. Some studies have shown that marine active peptides may improve the symptoms of colitis in vivo and in vitro [11,12,13]. Consuming natural, non-toxic, anti-inflammatory marine active peptides may establish an immune barrier, improving the body’s resistance to inflammation. This could become a novel and effective way to reduce the morbidity and prevalence of the disease. Low-molecular weight oyster peptides (LOPs) are produced by controlled enzymatic hydrolysis technology from oysters, which is mainly composed of small molecular peptides and fully retains the original vitamins, trace elements, taurine, and other nutrients found in oysters. As a result, LOPs are more easily and quickly absorbed by the human body than single amino acids or proteins [14]. Previous studies have shown that LOPs have biofunctional activities such as anti-oxidant [14,15,16], anticancer [17,18], immunomodulatory [19], and anti-inflammatory properties [16,20]. However, there is paucity in the literature on the prophylactic role of LOPs in acute colitis. LOPs have been shown to improve the structure of intestinal microbials [21]. Furthermore, many studies have confirmed the inhibitory effect of LOPs on pro-inflammatory factors [16,22,23]. Therefore, studying the preventive effects of LOPs on colitis is a promising strategy. 

Studies have shown that anti-inflammatory active peptides have a variety of modulatory effects on colitis, and their mechanisms include the modulation of inflammatory cytokine release and inflammation-related signalling pathways as well as the protection of the intestinal epithelial barrier and modulation of intestinal microbiota [24]. Lipopolysaccharide (LPS), a component of the cell wall of Gram-negative bacteria in the intestine, has been demonstrated to induce systemic inflammation and promote the release of pro-inflammatory factors through the intestinal barrier and through blood circulation [25]. In mice, acute severe colitis can be induced by intraperitoneal injection of LPS, which penetrates the intestinal wall. This is characterised by diarrhoea, weight loss, decreased anti-oxidant and anti-inflammatory capacity of colonic tissues, disruption in intestinal microbial balance, and low utilisation of short-chain fatty acids (SCFAs) in the intestine [26]. The feasibility of the LPS-induced colitis animal model is proven, providing an important reference point for our study [27,28,29]. 

In this study, an inflammation model of LPS-induced acute colitis in C57BL/6J mice was established by the addition of LOPs to the regular diet. The impact of LOP intervention on the acute colitis response was evaluated from multiple perspectives to elucidate the relevant mechanism, including colonic health, serum inflammatory factors, pathological conditions, intestinal microbiology, and short-chain fatty acids. The results of this study provide insight into the potential role of LOPs in the prevention of acute colitis in mice and offer a theoretical basis for further research.

## 2. Materials and Methods

### 2.1. Materials

The LOPs were obtained from Hainan SEMNL Biotechnology Co., Ltd. (Haikou, China). The enzyme-linked immunosorbent assay (ELISA) kits (Including IL-6, IL-1β and TNF-α) were obtained from Jiangsu Meimian Industrial Co., Ltd. (Yancheng, China); anti-oxidant test kits (T-SOD, CAT, GSH-Px, and MDA) were purchased from Nanjing Jiancheng Bioengineering Institute (Nanjing, China). LPS and high-purity SCFA standards were purchased from Sigma-Aldrich (St. Louis, MO, USA). A nitric oxide (NO) assay kit was obtained from Beyotime (Wuhan, China).

### 2.2. Analysis of the Basic Properties of Substances

The analysis of composition (carbohydrate, glycogen, peptides, amino acids, crude fat, ash, moisture, and energy) of LOPs was commissioned by Guangzhou GRG Metrologytest Co., Ltd. (Guangzhou, China). For analysis of the amino acid profile of LOPs, the freeze-dried LOPs, mixed with 6 mol L^−1^ HCl, were hydrolysed at 110 °C for 22 h and then analysed by an automatic amino acid analyser (S-433DUP, Sykam Chromatography, Munich, Germany). The composition of amino acids in the LOPs was expressed as g/100 g [19].

### 2.3. Peptides Sequencing

The LOPs were ultrafiltered (10 KD) and desalted for mass spectrometry, and the liquid phase used was 0.1% formic acid aqueous solution in liquid A and 0.1% formic acid/acetonitrile aqueous solution in liquid B (acetonitrile content was 84%). The liquid chromatography column (0.15 mm × 150 mm, RP-C18, Column Technology Inc., Fremont, CA, USA) was equilibrated with 95% liquid A. Samples were loaded from an autosampler onto peptide traps (Zorbax 300SB-C18,Agilent Technologies, Wilmington, NC, USA), separated on a liquid chromatography column, and analysed by mass spectrometry using a mass spectrometer (Q Exactive HF-X, Thermo Fisher Scientific Inc., Waltham, MA, USA).

### 2.4. Animals and Experimental Design

**Figure 1:**
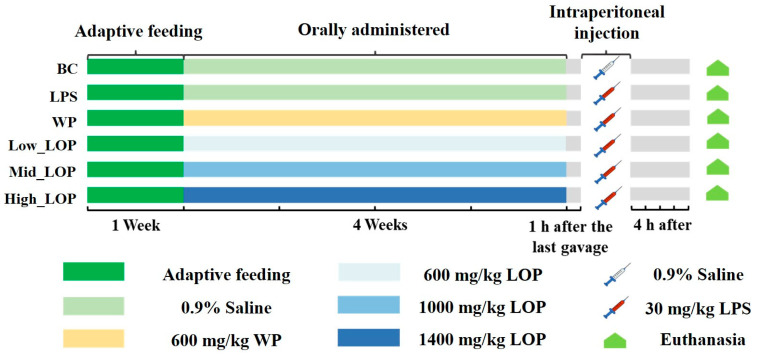
Animal experimental design.

The study employed a total of 42 male C57BL/6J mice of the SPF grade, which were of the specific pathogen-free variety (5 weeks old). The mice were purchased from ZhuHai Bestest Biotechnology Co., Ltd. (Zhuhai, China, SCXK<Guangdong>2020-0051). The experiments were conducted at the Animal Center at Guangdong Ocean University in accordance with the guidelines of the Experimental Animal Ethical Committees of Guangdong Ocean University (Permit No. GDOU-LAE-2023-013). Whey protein (WP), which has been demonstrated to inhibit lipopolysaccharide (LPS)-induced inflammatory responses [30], was employed as a positive control in this experiment. The experimental design and grouping are illustrated in Figure 1. After 1 week of acclimatisation, mice were randomly divided into 1 of 6 groups (*n* = 7 in each). Thereafter, mice in Low_LOP, Mid_LOP, and High_LOP groups were orally administered 600, 1000, and 1400 mg/kg bw per day, respectively, while the WP group was administered with WP at 600 mg/kg bw per day. Both the blank control (BC) group and LPS group were gavaged with equal volumes of saline. All animals were administrated with a basal diet and had free access to drinking water for 4 weeks in controlled environmental conditions at a temperature of 22 ± 1 °C, a normal humidity of 50–60%, and with a 12 h light/dark cycle.

One hour after the final oral administration, the LPS, WP, and three LOP groups received intraperitoneal injections of LPS at a dosage of 30 mg/kg body weight, while the BC group was administered an equivalent volume of saline via intraperitoneal injection. Mice were observed for diarrhea in the abdominal cavity at the anus, and the phenomenon was scored for diarrhea (DAI) 2 h after injection (the criteria are shown in Table S1). Four hours after the injection, whole blood was collected in EDTA-anticoagulated tubes. The residual blood samples were collected into 2 mL vacutainer tubes via the eyeball and subsequently centrifuged at 1000× *g* at 4 °C for 15 min to obtain serum. Following euthanasia, the colons of the mice were collected. A clean colon segment was fixed in a 4% paraformaldehyde solution for subsequent histomorphological observation. The faeces and residual colonic tissues were stored at a temperature of −80 °C.

### 2.5. Routine Blood Analysis

Blood cell counts, including the total number of white blood cells (WBCs), the ratio of lymphocytes (LYMs), and the platelet count (PLT), were performed using a haematology cell analyser (BV-5000Vet, Mindray Animal Medical Technology Co., Ltd., Shenzhen, China).

### 2.6. Serum Parameters Analysis

The detection of inflammatory factors in mice serum was performed using an enzyme-labelled instrument (Varioskan LUX, Thermo Fisher Scientific Inc., MA, USA). The concentrations of TNF-α, IL-1β, and IL-6 in the serum were measured using ELISA kits. The concentrations of nitric oxide were measured using a nitric oxide assay kit, and concentrations of IL-6, 1L-1β, and TNF-α were measured using ELISA kits.

### 2.7. Histopathological Observation

The colon tissue was subjected to an overnight paraformaldehyde soaking. Tissues embedded in paraffin were sectioned at a thickness of 5 μm and dewaxed. They were then stained with haematoxylin and eosin (H&E) [30]. A pathological examination of the colon was conducted using a Nikon microimaging system (Eclipse E100, Shanghai Nikon Instruments Co., Ltd., Shanghai, China).

### 2.8. Detection of Anti-Oxidant Properties

Measurement of anti-oxidant enzymes and MDA content was performed in colon by an enzyme-labelled instrument (Varioskan LUX, Thermo Fisher Scientific Inc., MA, USA). Colon tissues (20 mg) were mixed with saline (*w*/*v* = 1:9), and then ground and centrifuged at 10,000 (r/min) for 10 min at 4 °C. After the determination of tissue protein concentrations using the BCA method, the concentrations of T-SOD, CAT, GSH-Px, and MDA were determined by the kit.

### 2.9. Gut Microbiota Analysis

DNA was extracted and quantified using a nanodrop apparatus, and the quality of the extracted DNA was assessed by 1.2% agarose gel electrophoresis. In 25 μL of PCR product, add 0.8 times the volume of the magnetic beads and fully suspend them on a magnetic rack after adsorption for 5 min. Aspirate the supernatant. Then, add 20 μL of 0.8 times the volume of the magnetic beads washing solution and vibration. After adsorption for 5 min, the supernatant should be carefully aspirated. Then, 200 μL of 80% ethanol should be added, placed in the reverse direction on a magnetic rack, and fully adsorbed. The supernatant should then be absorbed. The tube should then be placed in room temperature and left for 5 min. After a five-minute interval, the alcohol should be allowed to evaporate completely. At this point, 25 μL of elution buffer should be added to elute the DNA. The PCR tube should then be placed on the adsorption rack for a further five minutes, after which the supernatant should be removed and transferred to a clean 1.5 mL centrifuge tube for storage. The products of the PCR amplification were subjected to fluorescence quantification, employing the Quant-iT PicoGreen dsDNA Assay Kit as the fluorescence reagent and a microplate reader (FLx800, BioTek Instruments Inc., Winooski, VT, USA) as the quantification instrument. The 16S rRNA gene sequencing analysis was performed by Suzhou PANOMIX Biomedical Tech Co., Ltd. (Suzhou, China). 

### 2.10. Faecal SCFAs Measurement

SCFAs were quantitatively analysed by a triple-quadrupole gas chromatograph mass spectrometer (GCMS-TQ8040NX, SHIMADZU, Kyoto, Japan). The mice faeces were accurately weighed at 300 mg and then homogenised with 100 μL of pre-cooled phosphate buffer in the presence of zirconia steel balls. Then, we added 1 mL of ethyl acetate and mixed thoroughly. The supernatants were collected after centrifugation at 4 °C for 3000 r/min for 10 min. Finally, the supernatant was passed through an organic membrane filter (0.22 μm). Finally, the SCFAs were quantified by a GC-MS analysis.

### 2.11. Statistical Analysis of Data

The results are expressed as the mean ± standard error of the mean (SEM). SPSS 27.0 software for Windows was used to perform an analysis of variance (ANOVA). Statistical significance was assessed by using one-way ANOVA followed by Tukey’s post hoc test. A *p* < 0.05 indicated significant difference. The graphs were drawn using Origin 2024. The graphical abstract was drawn using Figdraw 2.0.

## 3. Results and Discussion

### 3.1. Characteristic Analysis of the LOPs

The basic composition of the LOPs is listed in Table 1, where the main component is a peptide at a 67.2 g/100 g concentration followed by carbohydrate at a 22.1 g/100 g concentration.

Table 2 shows the analysis of the amino acid profile of the LOP. The ratio of essential to non-essential amino acids was 70.28%, indicating a higher concentration of essential amino acids in the LOP samples. Glutamic acid (10.2 g/100 g) was the most abundant amino acid in the sample, which may have immune system regulatory properties when consumed. Glutamate plays a crucial role in the nervous system, lymphocytes, and macrophages as an excitatory neurotransmitter and as a substrate for the formation of γ-aminobutyric acid. According to a recent review, peptides with immunomodulatory effects are typically composed of hydrophobic amino acids. The LOP sample was found to contain 39.34% hydrophobic amino acids and exhibited good hydrophobic activity. The amino acid composition and structure are similar to the amino acid composition of oyster peptides from Li et al. [19]. It can therefore be concluded that the LOPs are high-quality marine bioactive peptides.

### 3.2. Peptide Sequence

**Figure 2:**
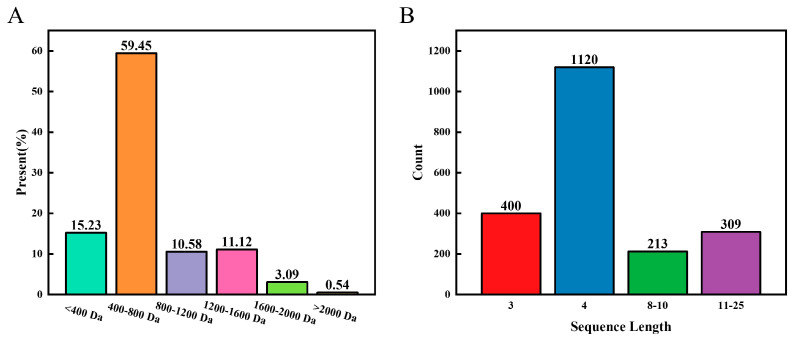
Peptide sequence of the LOPs: (**A**) peptide molecular weight distribution of LOP; (**B**) count of peptides of different lengths in the LOPs.

The structural composition of the LOPs was analysed by mass spectrometry. Figure 2A demonstrates the molecular weight composition of the peptides in the LOPs, with the most abundant peptides being 400 Da-800 Da and <400 Da, which accounted for 59.45% and 15.23% of the total. Among the identified peptides, tripeptides and tetrapeptides were the most abundant, with 400 and 1120 peptides (Figure 2B). In addition, Table S2 lists the amino acid sequences and molecular weights of all the peptides in the LOPs. Therefore, we can conclude that the LOPs are a substance dominated by low-molecular weight short peptides.

Low-molecular weight short peptides are able to participate in the regulation of various metabolic reactions in biological organisms and have been shown to have better anti-inflammatory activity [20,31,32]. In addition, the ability of peptides to resist enzymatic action and unfavourable pH conditions in organisms is affected by molecular weight; therefore low molecular weight LOPs are likely to have higher bioaccessibility [33]. In conclusion, the LOPs are therefore high-quality marine bioactive peptides.

### 3.3. Effects of the LOPs on the Basic Body Condition of Mice

**Figure 3:**
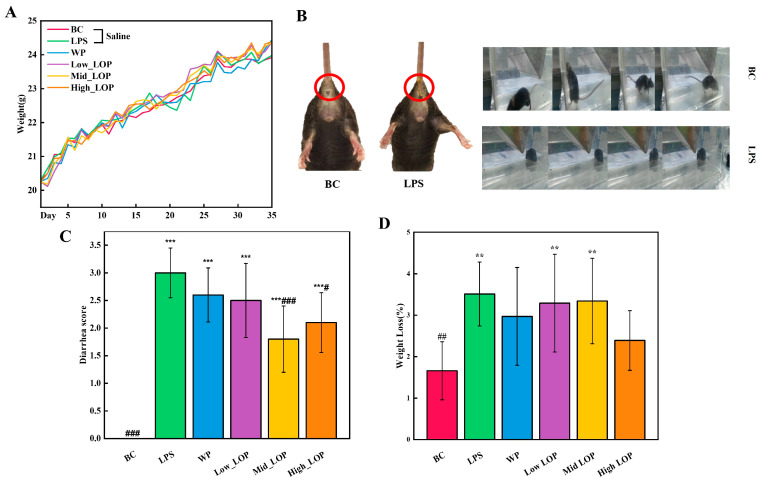
Experimental design and physiological characteristics of mice before and after intraperitoneal injection. (**A**) Changes in the body weight of mice in each group before intraperitoneal injection of LPS; (**B**) differences in faeces and behaviour between the BC and LPS groups of mice; (**C**) Diarrhea score; (**D**) weight loss. ** *p* < 0.01, *** *p* < 0.001 vs. the BC group; ^#^ *p* < 0.05, ^##^ *p* < 0.01, ^###^ *p* < 0.001 vs. the LPS group.

The changes in the body weight of mice before intraperitoneal injection of LPS are shown in Figure 3A, where the mice in the saline group had low body weights after 4 weeks of orally administered, whereas the mice in the WP and LOP groups had relatively high body weights; however, it is not significant (*p* > 0.05), suggesting that the oral administration of WP and LOP may have made the mice stronger and more resistant to adverse external factors [34].

In comparison with the BC group, mice that were injected intraperitoneally with LPS exhibited a markedly reduced range of motion, exhibited convulsive movements, and exhibited dishevelled hair on their backs. Additionally, they exhibited diarrhea and weight loss (Figure 3B). The LPS group exhibited the highest DAI score (Figure 3C), particularly in comparison with the other groups. These observations indicate that the injection of LPS induced a significant inflammatory response in the mice, thereby demonstrating the feasibility of LPS as a model of acute colitis. However, the WP and Low_LOP groups demonstrated improvement, although there was no significant difference (*p* > 0.05). In contrast, the Mid_LOP and High_LOP groups exhibited a significant improvement (*p* < 0.05). Furthermore, the LPS group exhibited a significant loss in weight, while both the WP and LOP groups demonstrated some improvement; however, none of these changes were found to be statistically significant (*p* > 0.05) (Figure 3D).

**Figure 4:**
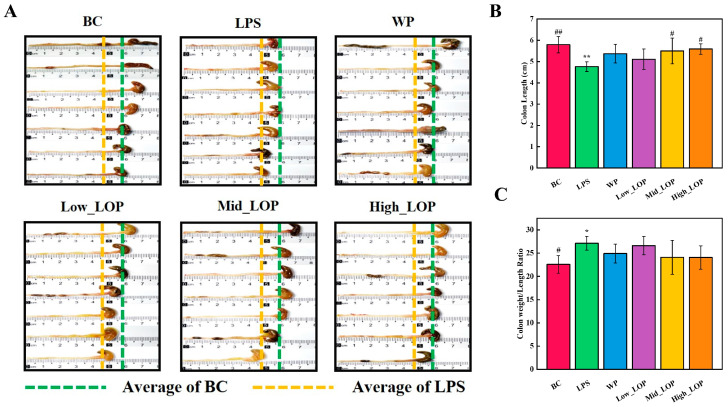
Colon condition (**A**–**C**). * *p* < 0.05, ** *p* < 0.01, vs. the BC group; ^#^ *p* < 0.05, ^##^ *p* < 0.01, vs. the LPS group.

The condition of the mice colon was showed in Figure 4. The average colon length of the BC group mice was the longest, while the colon of LPS group mice showed more obvious atrophy. The atrophies of Mid_LOP and High_LOP were significantly suppressed (*p* < 0.05). Additionally, the ratio of colon weight to length was found to correspond to the degree of colon oedema [35]. The LPS and Low_LOP groups had the highest ratio, indicating more severe colon oedema, while the WP, Mid_LOP, and High_LOP groups had lower ratios, suggesting some degree of oedema suppression, although there were no significant differences (*p* > 0.05).

The physiological traits of mice can provide a visual indication of their health status. However, biochemical indicators are still required to provide a more comprehensive assessment of their health.

### 3.4. Blood Routine

**Figure 5:**
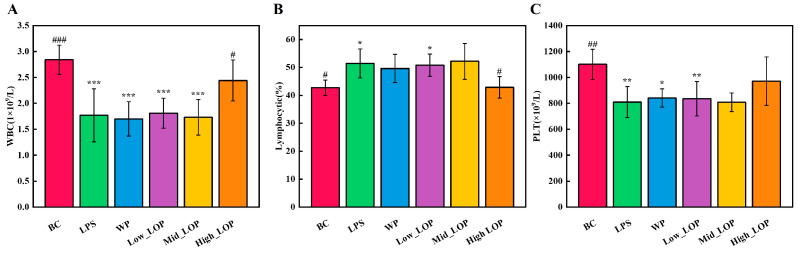
Blood routine (**A**–**C**). * *p* < 0.05, ** *p* < 0.01, *** *p* < 0.001 vs. the BC group; ^#^ *p* < 0.05, ^##^ *p* < 0.01,^###^ *p* < 0.001 vs. the LPS group.

By monitoring fluctuations in the number and distribution of blood cells in routine blood indicators, it is possible to ascertain the condition of the blood and identify potential disease. WBC, lymphocytes, and PLT are the most important indicators in routine blood in terms of diagnostics and reference values [36]. As shown in Figure 5, intraperitoneal injection of LPS caused significant changes in the above indices, further confirming the effectiveness of LPS injection modelling. 

The administration of LOPs ameliorated the significant abnormalities in WBC number, lymphocytes (%), and PLT number induced by LPS (Figure 5A–C) (*p* < 0.05), and the data showed that the High_LOP group exhibited a certain degree of alleviation for this abnormality, with a significant improvement in WBC number and lymphocytes (%) and no significant PLT number difference. However, the WP group and Low_LOP and Mid_LOP groups did not show significant improvement (*p* > 0.05). From a blood routine point of view, a specific dose of LOPs can be found to have an attenuating effect on the abnormal inflammatory response. However, further data are required in order to assess whether LOPs have an inhibitory effect on inflammation.

### 3.5. Anti-Inflammatory Effects of the LOPs

In the pathogenesis of colitis, dysregulation of the intestinal immune response represents a critical factor that may be accompanied by an imbalance in the expression of inflammatory factors [23]. The pro-inflammatory factors IL-6, TNF-α, and IL-1β promote early inflammatory responses and gradually amplify inflammation. They drive the inflammatory process by synergistically inducing the expression and production of other cytokines [37]. NO is produced primarily by the enzyme NOS-2 in many cell types involved in immunity and inflammation. It exerts complex regulatory activities in vitro and in vivo on the function, growth, and death of many immune and inflammatory cell types [38].

**Figure 6:**
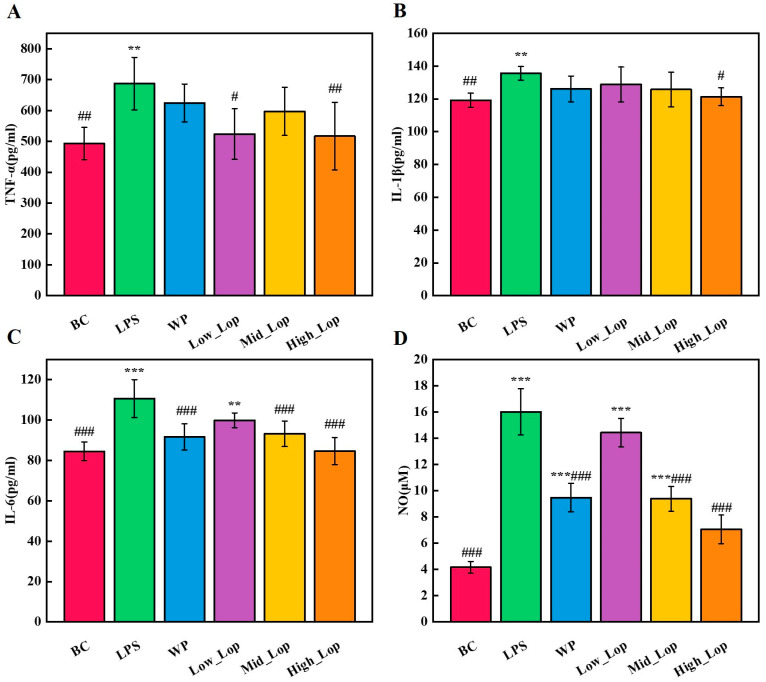
The concentration of the serum pro-inflammatory factors. (**A**) Concentration of TNF-α; (**B**) concentration of IL-1β; (**C**) concentration of IL-6; (**D**) concentration of NO. ** *p* < 0.01, *** *p* < 0.001 vs. the BC group; ^#^ *p* < 0.05, ^##^ *p* < 0.01, ^###^ *p* < 0.001 vs. the LPS group.

Figure 6A–D show the analysis of serum concentrations of pro-inflammatory factors, including TNF-α, IL-1β, IL-6, and NO. The serum concentrations of pro-inflammatory factors were significantly higher in the LPS group compared with the BC group (*p* < 0.05), particularly with respect to NO (*p* < 0.001). Changes in these indicators can further determine that the mice organism produced a significant inflammatory response after intraperitoneal injection of LPS. The WP group showed a significant inhibitory effect on IL-6 and NO compared with the LPS group (*p* < 0.001), but no significant inhibitory effect on TNF-α and IL-1β was observed. Both the Low_LOP group and the Mid_LOP group exhibited a good inhibitory effect. The High_LOP group significantly inhibited the concentrations of TNF-α, IL-1β, IL-6, and NO (*p* < 0.05), indicating a strong anti-inflammatory effect. In summary, the different dose groups of LOPs demonstrated some anti-inflammatory effects, which also showed concentration dependence in the IL-1β, IL-6, and NO levels. We can speculate that its anti-inflammatory effects may be related to the regulation of cellular inflammatory factor expression.

### 3.6. Histological Analysis

**Figure 7:**
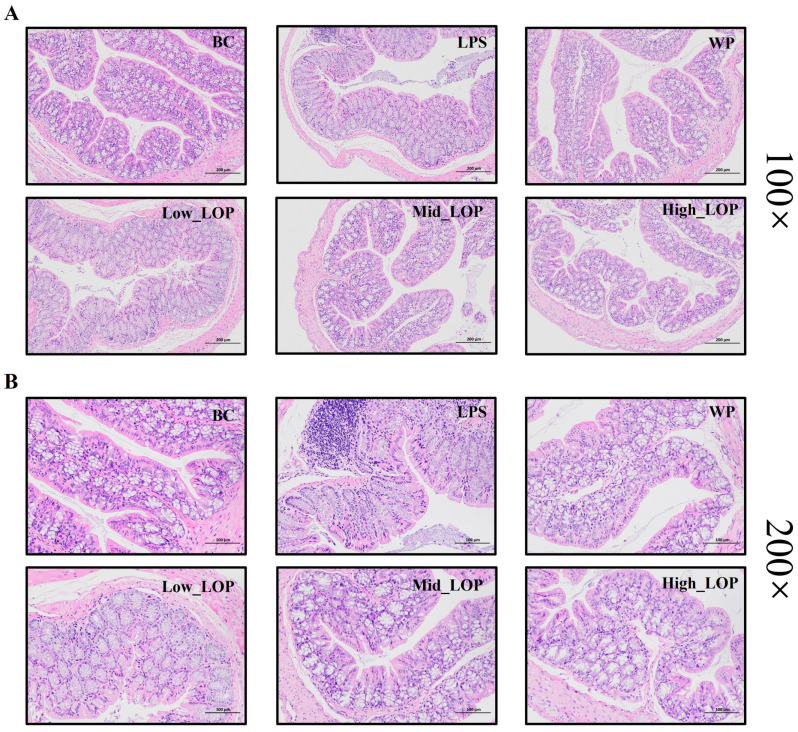
Representative histological images. (**A**) Mice colon tissue magnified by 100×; (**B**) mice colon tissue magnified by 200×.

Pathological sections allow for the visualisation of the health of the colon, the degree of tissue structural integrity, and the presence of inflammatory invasion. Figure 7 shows the results of HE staining. Figure 7A displays the 100× magnification of the H&E staining of the colon of mice in each group. The colon structure of mice in the BC group was normal, with regular crypt morphology, and the mucous membrane layer was intact. In contrast, the colon structure of mice in the LPS and Low_LOP groups was significantly damaged by inflammation, and the mucous membrane layer was visibly broken and detached. The destruction of the colon structure in mice was significantly suppressed in the WP group, with only a slight detachment of the mucous membrane layer. The overall structure of the colon in the Mid_LOP and High_LOP groups was slightly better than that of the LPS and Low_LOP groups. However, the mucosal layer remained detached in all two groups.

Figure 7B displays the colon of mice in each group at 200× magnification using H&E staining. The BC group mice exhibited undamaged goblet cell and well-arranged epithelial cells, with no apparent infiltration of inflammatory cells. This study found that the LPS group exhibited damage to the structure of the colon’s crypts and glands, with fewer or no goblet cells, disorganised epithelial cells, and localised inflammatory cell infiltration. The Low_LOP group also showed significant damage, while the normal mice had a better overall structure than the LPS and High_LOP groups, although the mucosal layer was still detached. The WP, Mid_LOP, and High_LOP groups exhibited a significantly higher number of goblet cells compared with the LPS group. Additionally, the crypts and glandular structures were more intact in these groups. These findings suggest that WP and LOPs may alleviate inflammatory injury to some extent. However, further analysis is still required.

### 3.7. Colon Tissue Anti-Oxidant Analysis

The body typically maintains a dynamic balance between the production and scavenging of free radicals. However, when this balance was disrupted, reactive oxygen species can accumulate and cause damage to the body or cells. Research has shown that several intestinal diseases are linked to reactive oxygen species or oxygen free radicals [39]. CAT, T-SOD, and GSH-PX are endogenous anti-oxidant enzymes in the body that function to scavenge reactive oxygen species. Therefore, the body’s ability to resist oxidative stress can be assessed by measuring the activity of these enzymes. In the presence of oxygen, free radicals and their active derivatives attack polyunsaturated fatty acids in cell membranes, leading to the generation of lipid peroxides that undergo a series of reactions to produce aldehydes such as MDA. As a result, the concentration of MDA can serve as an indirect indicator of the level of lipid peroxidation [40].

**Figure 8:**
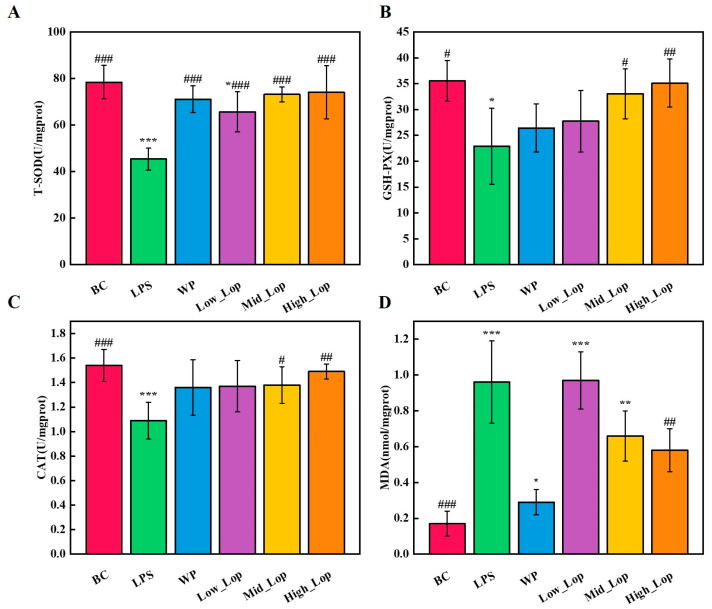
Concentrations of anti-oxidant indicators in mice colons. (**A**) T-SOD; (**B**) CAT; (**C**) GSH-Px; (**D**) MDA. * *p* < 0.05, ** *p* < 0.01, *** *p* < 0.001 vs. the BC group; ^#^ *p* < 0.05, ^##^ *p* < 0.01,^###^ *p* < 0.001 vs. the LPS group.

The concentrations of T-SOD, CAT, and GSH-Px in the colonic tissues of mice in the LPS group were reduced (*p* < 0.05), while the MDA content was significantly higher (*p* < 0.001) compared with the BC group, suggesting that LPS-induced inflammation reduces the anti-oxidant capacity of the colon (Figure 8). As shown in Figure 8A–C, T-SOD, CAT, and GSH-Px concentrations were higher in the WP group and in the different doses of the LOP groups compared with the LPS group, with no significant difference between the High_LOP groups and the BC group (*p* > 0.05). In addition, the High_LOP group had a significantly lower MDA content than the LPS group (Figure 8D). In this experiment, it can be found that LOPs can respond to inflammation by adjusting the level of oxidative stress. Specifically, LOPs not only inhibit lipid peroxidation but also scavenge excess superoxide anion radicals (O^2−^) and H_2_O_2_ in the body. MDA content was positively correlated with the level of lipid peroxidation. SOD scavenges O^2−^ in the body. CAT and GSH-Px protect the cells by removing H_2_O_2_. Thus, LOPs are not only a natural anti-inflammatory agent but also an anti-oxidant agent.

### 3.8. Effect of the LOPs on the Distribution of the Intestinal Flora

The intestinal flora represents a biological barrier within the intestinal tract. As an integral component of the intestinal barrier, it is involved in a multitude of vital physiological processes within the intestinal tract, including the maturation of the intestinal mucosal barrier function, the development of the immune system, nutrient transport and absorption, energy metabolism, and so forth [41]. When colitis occurs, it leads to changes in the intestinal microbial community, such as a decrease in the diversity of the microbiota and an increase in the ratio of harmful to beneficial bacteria. Therefore, we can further explore the mechanism of action of LOPs in protecting the colon from inflammation by studying the community diversity, microbial species, and distribution in faeces [42].

#### 3.8.1. Quantity and Length of the Sequence

The average sequence amount of the measured sample after DADA2 denoising was 32,383.94 (Table S3), and the sequence amount of each sample is shown in Table S4. The sequence length ranged from 232 bp to 442 bp, and the average length was 370.11 bp.

#### 3.8.2. Microbial Diversity Analysis

**Figure 9:**
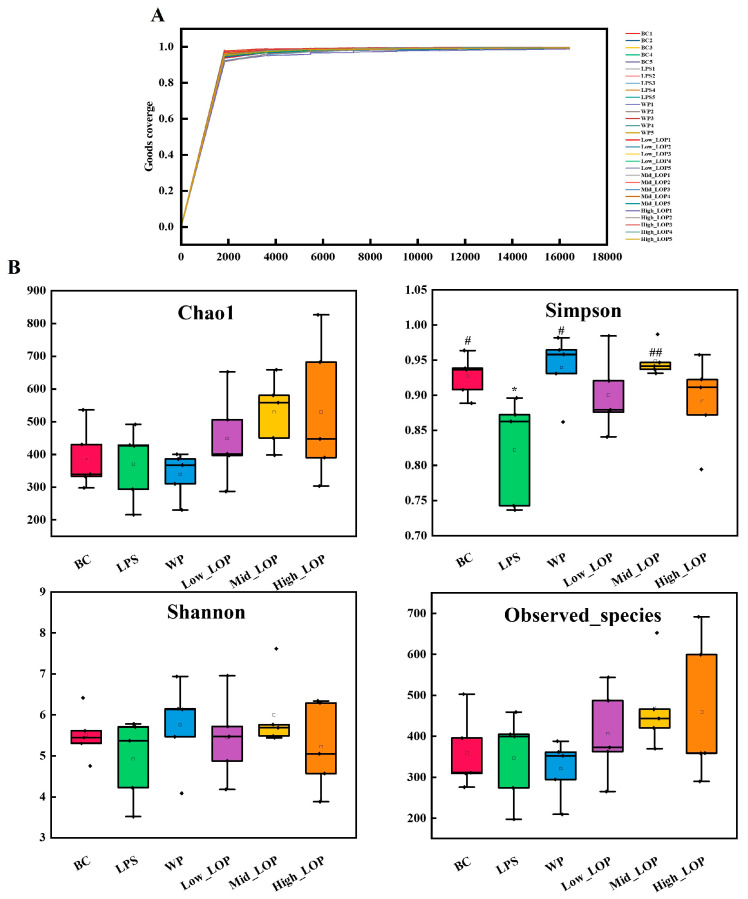
Diversity analysis. (**A**) Good’s coverge; (**B**) α diversity analysis. * *p* < 0.05, vs. the BC group; ^#^ *p* < 0.05, ^##^ *p* < 0.01, vs. the LPS group.

Indicators related to the biodiversity of the samples are shown in Figure 9. Chao1 was used to estimate the number of species actually present in the community, observed species was used to indicate the community’s species richness, the Shannon index integrates the richness and evenness of the community, the Simpson index was used to evaluate the diversity of the community, and Good’s coverage was used to assess the coverage of species in the community by sequencing.

As shown in Figure 9A, all sample data reached a plateau. The Good’s coverage index of all groups saturated the coverage of all sample species on the surface, indicating that the sequencing results were convincing. As can be seen in Figure 9B, compared with the normal group, the Chao1, Shannon, and observed species indices of the LPS group were reduced but not significantly different (*p* > 0.05), but the Simpson index was significantly reduced (*p* < 0.01). After LOP intervention, all indices of the Mid_LOP group were increased, especially with respect to the significant increase in the Simpson index. These findings indicate that LPS-induced colitis is associated with a reduction in the abundance and homogeneity of the intestinal flora in mice. Following the administration of WP and varying doses of LOPs, the mice exhibited improvements in all indicators.

#### 3.8.3. Microbial Composition Analysis

**Figure 10:**
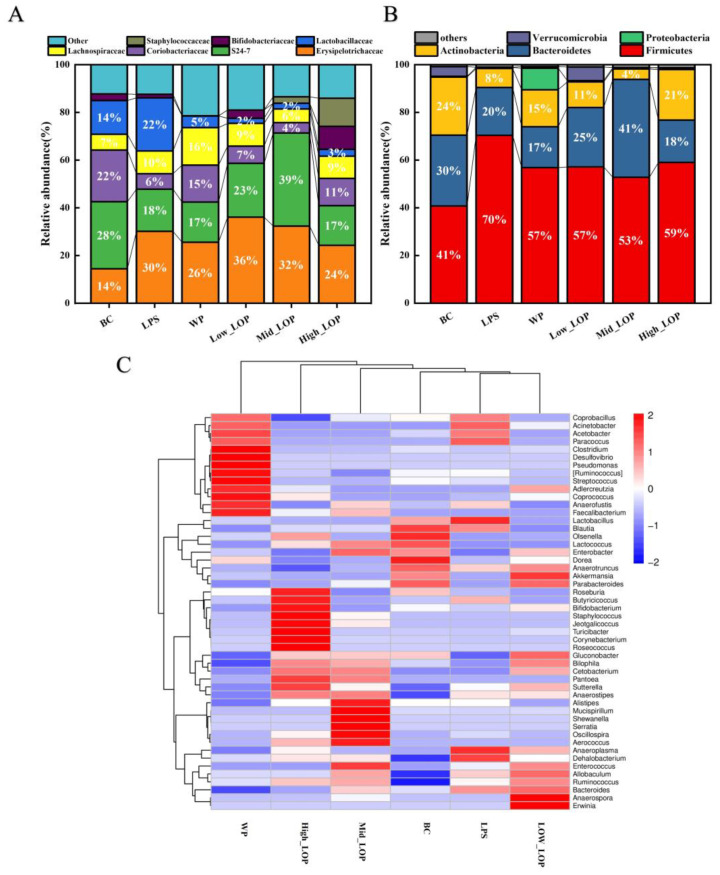
Structures of the intestinal microbial community in mice. (**A**) Intestinal microbial analysis of mice at the phylum level. (**B**) Intestinal microbial analysis of mice at the family level. (**C**) Cluster heat map of species abundance at the genus level.

To assess the effect of LOPs on the structure and composition of intestinal microbiota, the proportion of different species in each group was further analysed. At the phylum level, mice in all groups exhibited typical intestinal microbiota structures with the largest proportions being *Bacteroidetes*, *Firmicutes*, *Bacteroidetes*, and *Actinobacteria*, as illustrated in Figure 10A. In comparison with the BC group, the relative proportions of *Bacteroidetes* and *Actinobacteria* in the intestines of mice in the LPS group were reduced, while the relative abundance of *Firmicutes* was increased. The current state of knowledge regarding the impact of colitis on the *Firmicutes* phylum is inconclusive. However, it is evident that the *Firmicutes* phylum can exhibit either an abnormally high or low percentage [40,43,44]. Following oral administration of LOPs to mice, the Mid_LOP group exhibited a significant increase in the proportions of *Bacteroidetes* and *Firmicutes* relative to the BC group. Furthermore, the High_LOP group exhibited a significantly higher abundance of actinobacteria than the LPS group. These findings indicate that orally administered LOPs can alter the composition of gut microbial species at the phylum level, which may be a contributing factor to the enhanced preventive effect observed in the Mid_LOP and High_LOP groups.

At the family level (Figure 10B), the proportion of *Erysipelotrichaceae* significantly increased in mice in the LPS group. An abnormal increase in *Erysipelotrichaceae* tended to positively correlate with intestinal inflammation [45], while the High_LOP group showed some improvement. In contrast, the High_LOP group showed improvement. In addition, *S24-7* was positively correlated with the alleviation of intestinal inflammation [46], and the reduction of *S24-7* in the LPS group led to intestinal dysbiosis and diarrhea in the mice. Nevertheless, no reduction in the *S24-7* bacterial family was observed in the Low_LOP and Mid_LOP groups.

Figure 10C illustrates the temporal trend in the abundance distribution of the top 50 ASVs at the genus level within the sample. The sample and species distributions were clustered. It can be observed that there are notable differences between the LPS, Low_LOP, and BC groups. The LPS group exhibited a higher abundance of *Coprobacillus*, *Acinetobacter*, *Acetobacter*, and *Paracoccus*, which include a number of potentially harmful bacteria that can lead to septicaemia, genitourinary tract infections, and inflammatory diseases [47,48]. However, it is worth noting that after administering LOPs through oral administration, there was a significant decrease in the abundance of these particular genera. Furthermore, it was observed that the High_LOP group generated a greater number of beneficial genera, including *Bifidobacterium*, *Roseburia*, and *Butyricoccus*, compared with the BC group. For instance, *Roseburia* has been shown to impact various metabolic pathways [49], while *Bifidobacterium*, along with other anaerobes, can form a protective barrier on the intestinal mucosa, inhibiting the growth of pathogenic bacteria, regulating intestinal microbials and preventing diarrhea [50,51]. The Mid_LOP group also produced a higher amount of *Alistipes*, *Mucispirillum*, and *Oscillospira*, among other beneficial bacteria. For instance, *Mucispirillum* can antagonise *Salmonella* virulence to protect mice against colitis as well as regulate the immune system [52].

Scientific studies have shown that the greater the abundance of a community, the more likely it is to resist sudden, unfavourable environmental changes. Our results showed that both WP and LOPs increased the diversity of the colonic microbiota and effectively improved the ratio of beneficial to harmful bacteria, so we can speculate that one of the mechanisms by which LOPs protect the colon from inflammation is that LOP intake optimises the structure of the intestinal community in mice colons.

### 3.9. Faecal SCFAs Production

SCFAs are metabolites produced by the intestinal microbial during carbohydrate catabolism. They can bind to specific receptors and exert regulatory effects. Following the production of SCFAs in the intestine, a small portion of it directly crosses the epithelial cell barrier in bound form. Another small portion of propionic acid and acetic acid is transported to the liver and participates in the tricarboxylic acid cycle, where it is metabolised to glucose and provides energy. The majority of SCFAs exist in the free form and regulate the body’s stress response by binding to specific receptors. The remaining SCFAs are excreted with the faeces and through other pathways [53]. Consequently, the content of SCFAs in mouse faeces can be employed to infer the degree of utilisation of SCFAs in the intestine and in the health status of mice.

**Figure 11:**
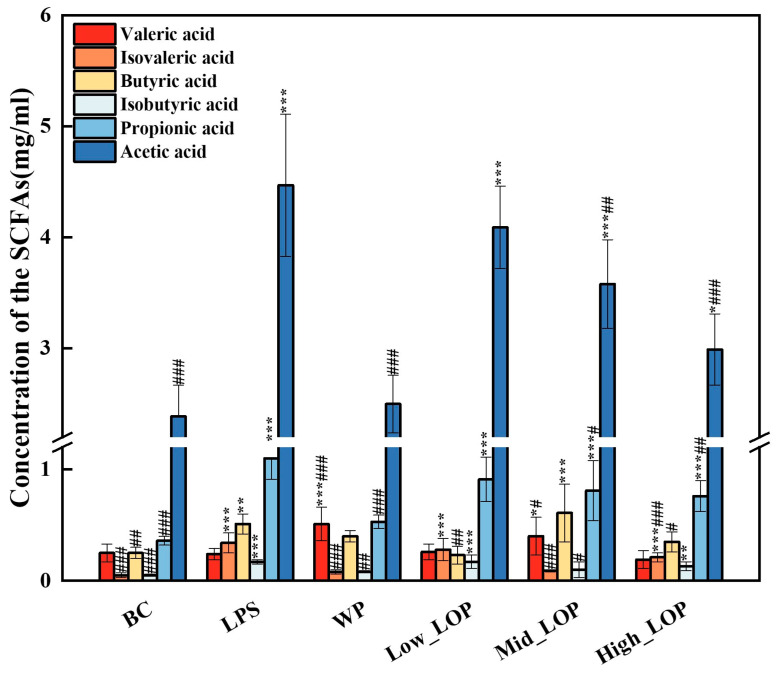
Concentration of the SCFAs in the colon content of mice (*n* = 6). * *p* < 0.05, ** *p* < 0.01, *** *p* < 0.001 vs. the BC group; ^#^ *p* < 0.05, ^##^ *p* < 0.01, ^###^ *p* < 0.001 vs. the LPS group.

Figure 11 shows the SCFA content in the faeces of each group. The contents of acetic acid, propionic acid, butyric acid, and isobutyric acid in the faeces of mice in the model group were significantly higher (*p* < 0.01) than those in the normal group. Compared with the model group, the WP group showed a significant decrease in acetic acid, propionic acid, isobutyric acid, and isovaleric acid (*p* < 0.01). In all three groups of LOP intervention, acetic acid decreased, and propionic acid and isovaleric acid significantly decreased in the Mid_LOP and High_LOP groups (*p* < 0.05), The findings were consistent with those of Han et al. [54]. In combination with the results of colonic histopathology and microbial composition, it can be hypothesised that LOPs have a protective effect on the intestinal structure and that the increased abundance of beneficial bacteria promotes a more efficient absorption of SCFAs by the intestinal tract, which in turn participates in the body’s circulation and plays a positive role. Excess SCFAs will be excreted in the faeces, thus reducing the amount of SCFAs in the faeces. Therefore, the intake of LOPs is likely to cause an increase in the number of beneficial bacteria in the intestinal tract, which indirectly promotes the utilisation of short-chain fatty acids, thus further protecting the colon tissue from inflammation.

## 4. Conclusions

In this study, LOPs protected the mice colon from inflammation caused by LPS. The oedema condition, atrophy, was suppressed, and the colonic tissue structure was protected. It also had a significant preventive effect on LPS-induced acute colitis in mice by inhibiting the production of pro-inflammatory cytokines and improving the anti-oxidant capacity of the intestine. In addition, through high-throughput sequencing, we speculated that LOPs could promote intestinal absorption and the utilisation of short-chain fatty acids by altering the abundance of intestinal microorganisms and increasing the proportion of beneficial bacteria. The alteration of the intestinal flora distribution is likely to be one of the mechanisms by which LOPs protect the colon against inflammation. However, the pathogenesis of colitis is a complex process, and our study has not yet elucidated the complete mechanisms in greater detail. In future studies, greater attention should be paid to scientific questions such as whether LOPs are involved in influencing the role of tight-junction proteins (ZO-1, occludin) of colonic tissues in the intestinal barrier, the predominant bacterial genera involved in the prevention of colitis, and the ways in which they are involved in the immune process and inflammatory signalling pathways (NF-κB, MAPK) in a scientific manner.

## Figures and Tables

**Table 1:** Composition of the LOPs.

	Low Molecular Weight Oyster Peptides g/100 g (or KJ)
Carbohydrate	22.10
Peptides	67.20
Crude fat	2.00
Ash	4.90
Moisture	5.83
Energy	1518.00 KJ

**Table 2:** Amino acid composition of the LOPs.

Amino Acid	Quantity in the LOPs (g/100 g)
Glutamate	10.2
Threonine	3.06
Serine	2.97
Proline	3.43
Glycine	3.81
Alanine	3.44
Valine	3.00
Methionine	1.30
Isoleucine	3.00
Leucine	4.04
Tyrosine	2.03
Phenylalanine	2.05
Histidine	1.13
Lysine	5.64
Arginine	4.97
Aspartate	7.11
The percentage of hydrophobic amino acids (%)	39.50%
Ratio of essential amino acids to non-essential amino acids (%)	70.28%

## Data Availability

The original contributions presented in the study are included in the article/Supplementary Materials, further inquiries can be directed to the corresponding author.

## Supplementary Materials

The following supporting information can be downloaded at: https://www.mdpi.com/article/10.3390/foods13152391/s1, Table S1: The DAI score criteria; Table S2: Amino acid sequence and molecular weight of LOP; Table S3: Sequencing quantity of each sample; Table S4: The counts of sequence length
